# Supplementary material for: Autocrine Wingless constricts the Drosophila embryonic gut by Ca+2-mediated repolarisation of mesoderm cells
Source: EMBO Rep. 2025 Mar 7;26(7):1737–48. doi: 10.1038/s44319-025-00411-x (PMC11977022; doi:10.1038/s44319-025-00411-x)
Supplement: Supplementary file 2 — Movie EV1 [file 44319_2025_411_MOESM2_ESM.zip › Movie EV1/Figure legend movie EV1.docx]

**Movie EV1**. Time laps imaging of 24B*>GCamP* embryo from early stage 15 to late stage 16 showing the Ca^2+^ pulses associated with the middle, the posterior and anterior constrictions. Anterior side of the embryo is on the left. Images were aquired each over 60 s over 50-75 µm.
