# Supplementary material for: Autocrine Wingless constricts the Drosophila embryonic gut by Ca+2-mediated repolarisation of mesoderm cells
Source: EMBO Rep. 2025 Mar 7;26(7):1737–48. doi: 10.1038/s44319-025-00411-x (PMC11977022; doi:10.1038/s44319-025-00411-x)
Supplement: Supplementary file 3 — Movie EV2 [file 44319_2025_411_MOESM3_ESM.zip › Movie EV2/Figure legend Movie EV2.docx]

**Movie EV2**. Time laps imaging of *ClC-a ^-/-^*; 24B*>GCamP* embryo from early stage 15 to late stage 16 showing the absence of the Ca^2+^ pulses. Anterior side of the embryo is on the left. Images were acquired each over 60 s over 50-75 µm.
