# Supplementary material for: Autocrine Wingless constricts the Drosophila embryonic gut by Ca+2-mediated repolarisation of mesoderm cells
Source: EMBO Rep. 2025 Mar 7;26(7):1737–48. doi: 10.1038/s44319-025-00411-x (PMC11977022; doi:10.1038/s44319-025-00411-x)
Supplement: Supplementary file 4 — Expanded View Figures [file 44319_2025_411_MOESM4_ESM.pdf]

Expanded View Figures

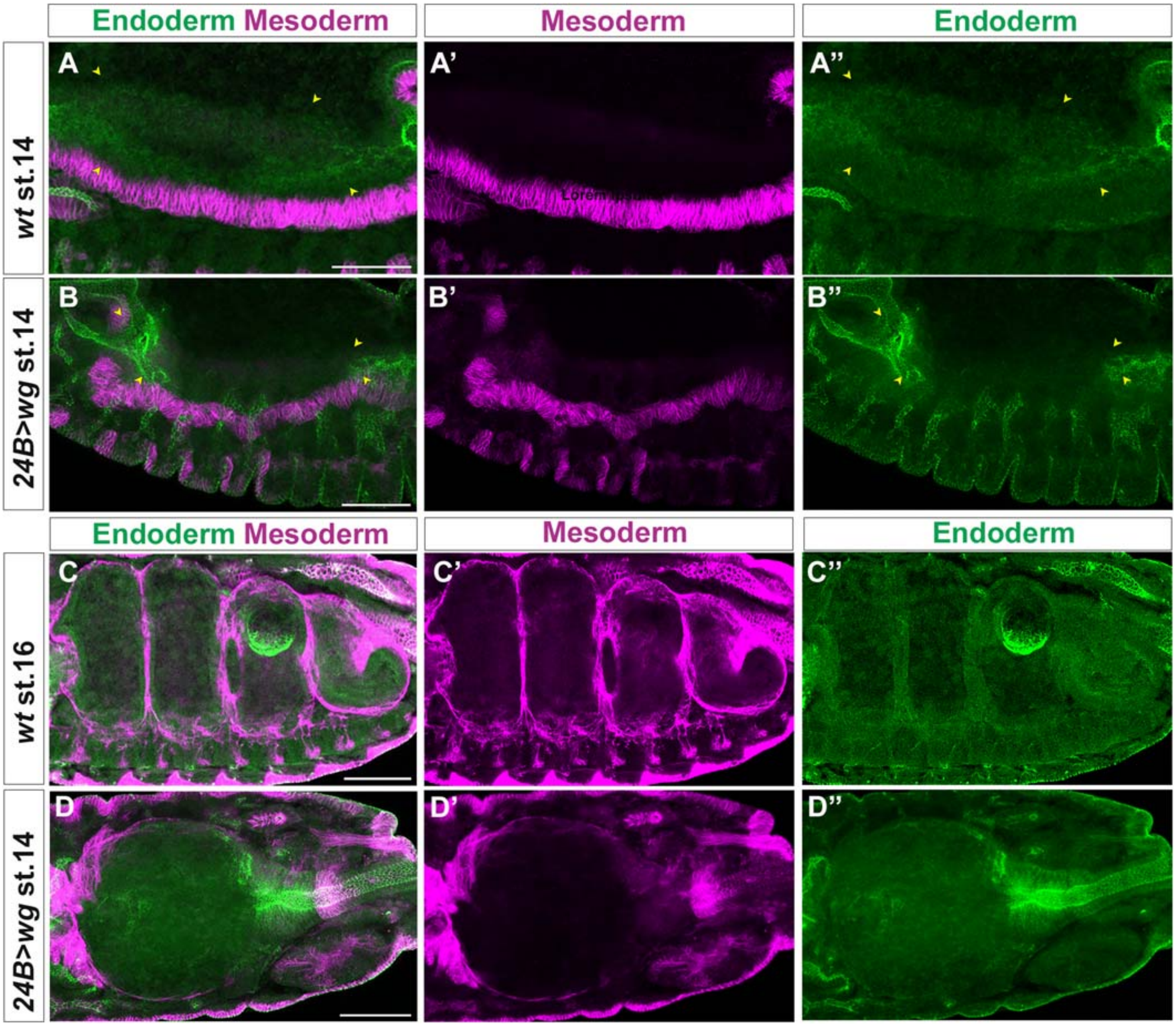

Figure EV1. Overexpression of wingless in the visceral mesoderm, hinders endoderm migration.

(**A–D**) Midgut of wild-type (**A**, **C**) and 24B>*wg* (**B**, **D**) embryos at stage 14th (**A**, **B**) and stage 16th, (**C**, **D**) stained with antibodies against FasIII in magenta and against DECadherin in green. Upon *wg* overexpression (**B**), the row of mesodermal cell does not properly form and the endodermal tissue does not migrate correctly (arrowheads). At later stages, *wg* overexpressing midguts (**D**) completely lose their organization. Anterior is left, dorsal is top, scale bar 50 μm.

 © The Author(s)

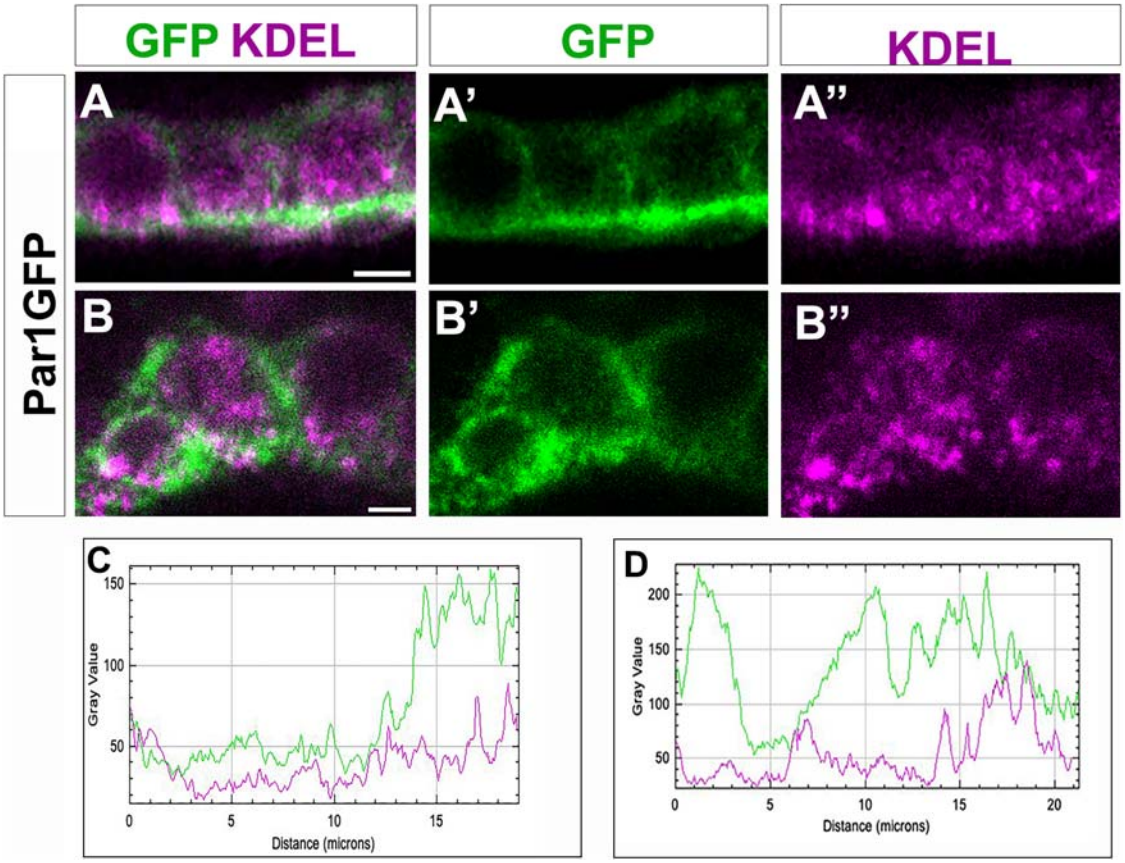

Figure EV2. Non-uniform localization of Par-1.

(**A**, **B**) Non-constricting (**A**) and constricting (**B**) visceral mesodermal cells of a *par1-GFP* embryo stained with GFP (green) and KDEL (magenta) to visualize the ER in the cytoplasm. Internal membrane is up, Scale bar 2 μm. (**C**, **D**) Plots showing the differential distribution of Par-1 (green line) and KDEL(magenta line) intensity in visceral mesoderm cells away from the constriction (**C**) and in constricting visceral mesodermal cells (**D**).

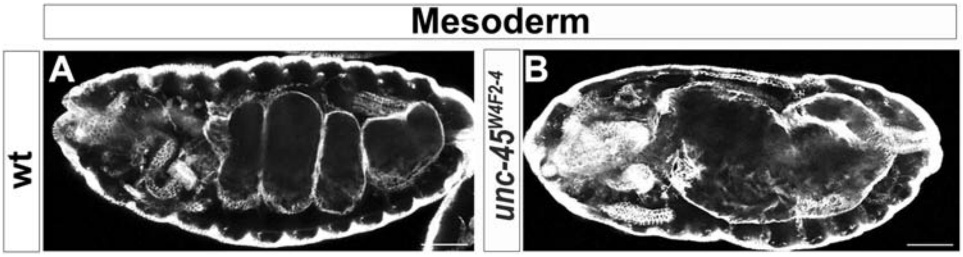

Figure EV3. *unc-45* mutant embryos show a midgut constriction failure.

Wt (**A**) and *unc-45*$^{4F2-4}$(**B**) embryos at stage 16th labelled with antibodies against FasIII to visualise the visceral mesoderm. Embryos are shown in a lateral view, anterior to the left, scale bar 50 μm.

 © The Author(s)
